# Hand-to-Hand Model for Bioelectrical Impedance Analysis to Estimate Fat Free Mass in a Healthy Population

**DOI:** 10.3390/nu8100654

**Published:** 2016-10-21

**Authors:** Hsueh-Kuan Lu, Li-Ming Chiang, Yu-Yawn Chen, Chih-Lin Chuang, Kuen-Tsann Chen, Gregory B. Dwyer, Ying-Lin Hsu, Chun-Hao Chen, Kuen-Chang Hsieh

**Affiliations:** 1 Sport Science Research Center, National Taiwan University of Sport, Taichung 40404, Taiwan; sk.lu2002@gmail.com; 2 Department of Hospitality, Recreation, and Tourism Management, East Stroudsburg University of Pennsylvania, East Stroudsburg, PA 18301, USA; chiang1999@gmail.com; 3 Department of Physical Education, National Taiwan University of Sport, Taichung 40404, Taiwan; yu11.tw@yahoo.com.tw; 4 Department of Radiology, Jen-Ai Hospital, Taichung 41265, Taiwan; xray@mail.jah.org.tw; 5 Department of Applied Mathematics, National Chung Hsing University, Taichung 40227, Taiwan; chen508@gmail.com (K.-T.C.); ylhsu@nchu.edu.tw (Y.-L.H.); 6 Department of Exercise Science, East Stroudsburg University of Pennsylvania, East Stroudsburg, PA 18301, USA; gdwyer@po-box.esu.edu; 7 Office of Physical Education, Tunghai University, Taichung 40704, Taiwan; haogochen@gmail.com; 8 Office of Physical Education and Sport, National Chung Hsing University, Taichung 40227, Taiwan; 9 Research Center, Charder Electronic Co., Ltd., Taichung 41262, Taiwan

**Keywords:** anthropometrics, estimate equation, fat free mass (FFM), hand-to-hand (HH), standing position

## Abstract

This study aimed to establish a hand-to-hand (HH) model for bioelectrical impedance analysis (BIA) fat free mass (FFM) estimation by comparing with a standing position hand-to-foot (HF) BIA model and dual energy X-ray absorptiometry (DXA); we also verified the reliability of the newly developed model. A total of 704 healthy Chinese individuals (403 men and 301 women) participated. FFM (FFM_DXA_) reference variables were measured using DXA and segmental BIA. Further, regression analysis, Bland–Altman plots, and cross-validation (2/3 participants as the modeling group, 1/3 as the validation group; three turns were repeated for validation grouping) were conducted to compare tests of agreement with FFM_DXA_ reference variables. In male participants, the hand-to-hand BIA model estimation equation was calculated as follows: FFM^m^_HH_ = 0.537 h^2^/Z_HH_ − 0.126 year + 0.217 weight + 18.235 (*r*^2^ = 0.919, standard estimate of error (SEE) = 2.164 kg, *n* = 269). The mean validated correlation coefficients and limits of agreement (LOAs) of the Bland–Altman analysis of the calculated values for FFM^m^_HH_ and FFM_DXA_ were 0.958 and −4.369–4.343 kg, respectively, for hand-to-foot BIA model measurements for men; the FFM (FFM^m^_HF_) and FFM_DXA_ were 0.958 and −4.356–4.375 kg, respectively. The hand-to-hand BIA model estimating equation for female participants was FFM^F^_HH_ = 0.615 h^2^/Z_HH_ − 0.144 year + 0.132 weight + 16.507 (*r*^2^ = 0.870, SEE = 1.884 kg, *n* = 201); the three mean validated correlation coefficient and LOA for the hand-to-foot BIA model measurements for female participants (FFM^F^_HH_ and FFM_DXA_) were 0.929 and −3.880–3.886 kg, respectively. The FFM_HF_ and FFM_DXA_ were 0.942 and −3.511–3.489 kg, respectively. The results of both hand-to-hand and hand-to-foot BIA models demonstrated similar reliability, and the hand-to-hand BIA models are practical for assessing FFM.

## 1. Introduction

Obesity-associated metabolic syndrome and diabetes are becoming more prevalent across all ages and ethnic groups worldwide [1]. Fat free mass (FFM) is a very important concept in studying body composition. The FFM is the difference between total body mass and fat mass (FM). In participants or patients, the FFM or FM is a valid and reliable evaluation method for determining the use of nutrient supplements, prevention of muscle dystrophy, and obesity. The existing anthropometric measurement methods for evaluating body composition, including body mass index (BMI), skinfold thicknesses, and body circumference, have limitations. The ideal body composition methods should be low-cost, non-invasive, convenient for subjects, easy to perform by non-skilled personnel, and capable of generating repeatable and accurate results [2]. Hand-to-hand bioelectrical impedance analysis (BIA) is considered to be the most convenient BIA method, and has been in use for over a decade. However, its accuracy has not been reported in certain ethnic populations, such as Asians.

To validate the accuracy of BIA methods, some studies have shown excellent agreement between BIA and dual energy X-ray absorptiometry (DXA)-derived measurements across the general population [3]. In addition, the hand-to-foot BIA model has shown great accuracy in the measurement of fat free mass using tetra-polar gel impedance electrodes [4]. The use of the hand-to-foot BIA model has primarily been limited to laboratories, and has not been used for commercial use due to its more complex and costly testing procedures. Regarding the hand-to-hand BIA model, previous studies have delivered inconsistent results in validating its accuracy and precision [5].

Previous studies have reported the use of BIA impedance measurements taken in the supine position, but only a few studies have used the standing position [6,7,8]. While the hand-to-hand BIA model reportedly has a high reference value [9], hand-to-hand model BIA-estimated body fat percentage (BF%) significantly underestimates the reference value [6,10]. Therefore, the current ability of the hand-to-hand model to predict BF% has varied. In addition to the measuring position, differences in the configuration, electrode location, electrode materials, and contact area can affect measurement consistency and accuracy [11].

The standing hand-to-hand BIA model is convenient. The BIA FFM estimation equation needs to refer to a particular reference method that is established based on a representative population. After confirmation, the estimation equation may be a viable tool for estimating BIA FFM. In many studies, BIA FFM estimation equations have mainly used dual-energy X-ray absorptiometry (DXA), hydrodensitometry, and total body potassium as reference or confirming methods. In the present study, we used DXA as the reference method to verify a healthy Chinese population with a random sampling method. Few studies have undertaken this research, especially in the Chinese population. In addition, the results of the hand-to-hand and hand-to-foot BIA model were compared to evaluate the accuracy of these two models.

## 2. Materials and Methods

### 2.1. Participants

**Figure 1:**
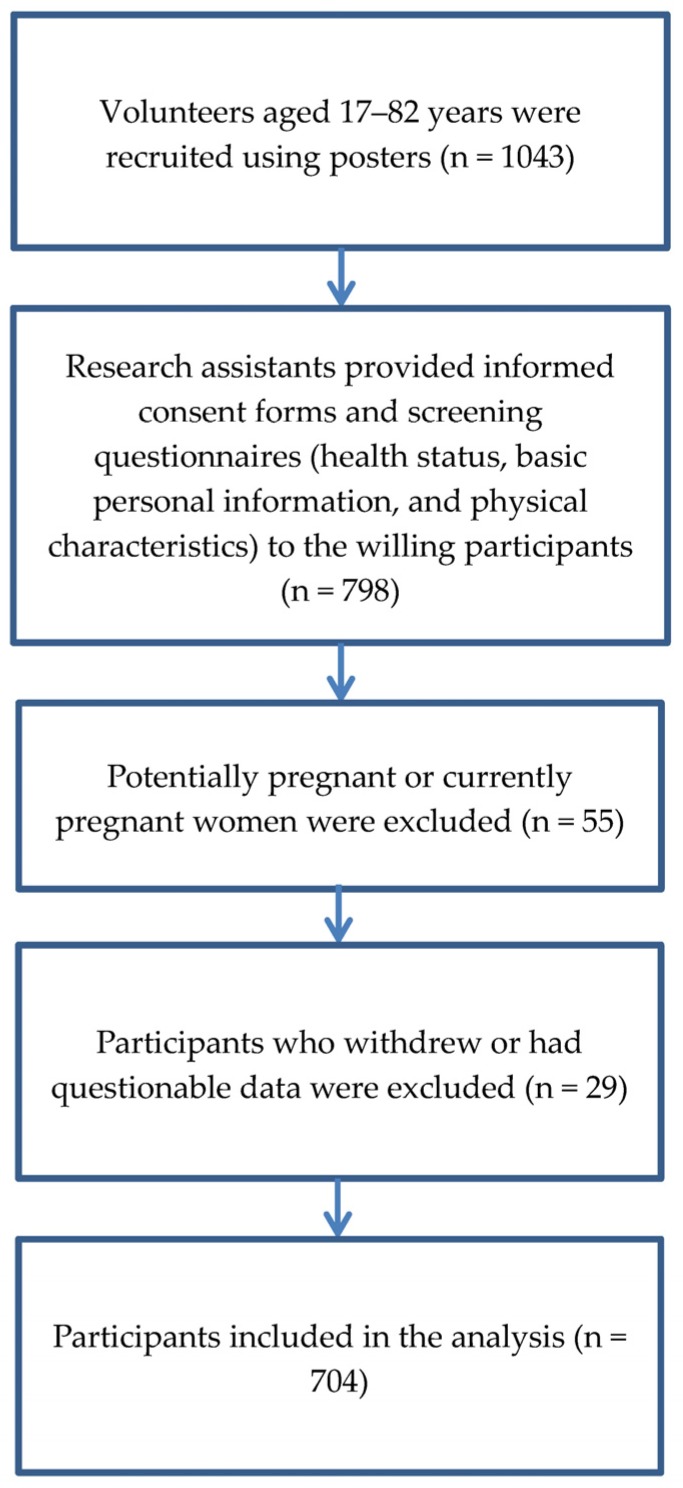
Flow chart of the study participants included in the analysis.

Healthy adults of Asian ethnicity with no recent history of alcohol consumption, tobacco use, or hospitalization were included (Figure 1). Subjects with one or more of the following conditions were excluded: hypertension, diabetes mellitus, cancer, renal failure, hepatitis-related diseases, chronic pulmonary diseases, pregnancy, or any artificial electrical implantation. In addition, persons with a disability, currently menstruating, administered hormones, with body shape abnormalities, and children were excluded.

The subjects were recruited after providing informed consent under the permission (IRB 97-01) of the Institutional Review Board (IRB) of the Advisory Committee at Jen-Ai Hospital in Taiwan. Individual subjects were informed of the experimental purpose, methods, procedures, and safety-related information. A nonrandom purposive sampling approach was adopted to recruit subjects with a wide range of weights, heights, and sexes ranging from 17 to 82 years old across central Taiwan. They were recruited by advertisement and by contacting societies.

### 2.2. Impedance Measurements

**Figure 2:**
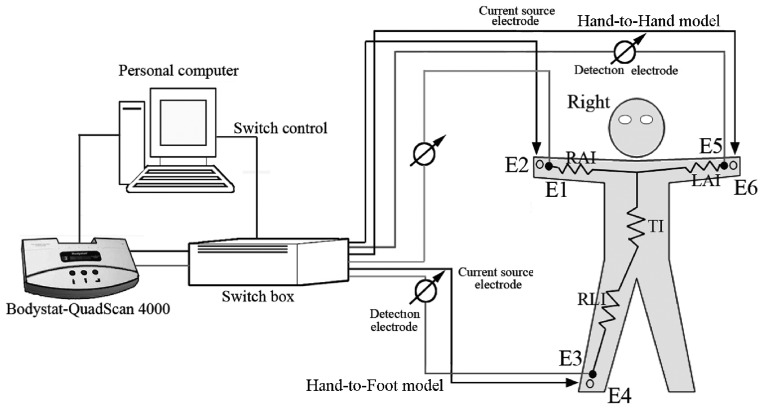
Impedance measurement system. E1, E3, and E5 were measuring electrodes, and E2, E4, and E6 were current electrodes. After forming the circuit between E2 and E4, the measurement of E1 and E3 yielded the hand-to-foot impedance: RAI + TI + RLI (Z_HF_). Similarly, the circuit between E2 and E6 and the measurement of E1 and E5 yielded the hand-to-hand impedance: RAI + LAI (Z_HH_). RAI, right arm impedance; TI, trunk impedance; LAI, left arm impedance; RLI, right leg impedance.

The purpose of the impedance measurement was to analyze the electrode values that passed through the upper right segment, trunk, and lower segments of the body. For these measurements, we used the Quadscan 4000 machine (Bodystat, Isle of Man, Douglas, UK), which can be switched between hand-to-foot and hand-to-hand impedance measurements using a connected computer. The instrument and measurement model are shown in Figure 2. Frequencies of 5, 50, 100, and 200 kHz are available through the Quadscan 4000. A frequency of 50 kHz was used in the hand-to-hand and hand-to-foot models in a standing position for all subjects, in order to confirm that the BIA measurements were consistent and that no changes would occur, regardless of time and different postures.

E1, E3, and E5 were the measuring electrodes, and E2, E4, E6 were the current electrodes. E1, E2, E5, and E6 are located on the handle, and E3 and E4 are located on the right side of the platform. The impedances measured in each body segment in a standing position were labeled as follows: RAI, right arm impedance; TI, trunk impedance; LAI, left arm impedance; and RLI, right leg impedance. After forming the circuit between E2 and E4, the measurement of E1 and E3 hand-to-foot impedance yielded RAI + TI + RLI (Z_HF_, unit: ohms). Similarly, the circuit between E2 and E6 and the measurement of E1 and E5 hand-to-hand impedance yielded RAI + LAI (Z_HH_ unit: ohms). All measurements were performed in a climate-controlled laboratory at 25 °C room temperature and 75% relative humidity.

The Quadscan 4000 electrode wires were connected through a channel switch box controlled by the computer. The switch box was further connected to the E1, E3, and E5 stainless steel electrode measuring plate and E2, E4, and E6 stainless steel current electrode plates. An Omron HBF-361 (Omron Healthcare, Kyoto, Japan) platform was used as the impedance measuring base, and included handles, a stainless steel polar plate, and a cable. The electrical stainless steel area of the handle and base were 4 × 9 cm^2^ and 5 × 9 cm^2^, respectively. Further, we collaborated the direct measures from the stainless steel electrodes and circuit switch using the impedance collaborator provided by the Quadscan 4000 manufacturer. The impedance measures were 496–503 ohms, and no significant differences were found between the two methods.

We used the detector electrodes (E1, E3) and current source electrodes (E2, E4) to form a measurement circuit. After measuring hand-to-foot model impedance, we canceled the E3, E4 function located at the lower right foot and activated the E5, E6 at the right handle and retained the E1, E2 function, forming another measurement loop to obtain the hand-to-hand impedance model. Both hand-to-foot or whole body models were created by adding the arm, trunk, and leg impedance values together.

The within- and between-day coefficients of variation (CVs) (%, standard deviation (SD)/mean) of the impedances were measured and calculated, respectively, through right-hand to right-foot and right-hand to left-hand pathways to ensure the repeatability of the impedance measurements. Impedance measurements were performed with six subjects (three men, three women) on five consecutive days to estimate the between-day CV. The impedance was then measured ten times for each subject within an hour on the same day to estimate the within-day CV.

### 2.3. Experimental Procedures

Subjects wore cotton medical gowns without any metal attachments. Body weight, height, and whole body DXA (Lunar Prodigy Advance, GE Healthcare, Madison, WI, USA) using enCore 2003 Version 7.0 software (Madison, WI, USA, 2004) were measured for all subjects. BF% and FFM were measured using DXA as BF%_DXA_ and FFM_DXA_, respectively. Then, for the impedance measurements, subjects stood on a platform embedded with bi-polar electrodes and gripped the handles embedded with the tetra-polar electrodes. Subjects kept their arms straight, holding the handle with their hand while maintaining a 90-degree angle with the trunk. No alcoholic beverages were consumed within the previous 48 h, no diuretic was administered in the previous 7 days, and urination had not occurred within 30 min prior to the BIA and DXA measurements. None of the subjects reported a body weight variation >5 kg throughout the four-month period.

The calibration standard tests the mechanical operation and calibration of the DXA machine. Machines perform a continuous internal calibration with each pixel measurement by automatically using a rotating internal calibration wheel or drum. The instrument calibration process requires five consecutive phantom measurements with repositioning. The mean value should differ by <1% from the manufacturer-provided value.

### 2.4. Statistical Analysis

The results are presented as the mean ± SD. We determined that we needed a minimum sample size of 119 or 129 subjects for three or four estimate variables, respectively, using an effect size of 0.15 (*f*^2^, medium), a 0.05 probability of error, and a power of 0.95 (1 − β error probability) [12]. Linear regression analyses were used, with the stepwise method, to develop the prediction equations for estimating FFM in both male and female subjects. FFM_DXA_ was the dependent variable, while age, sex, body weight, height, and bioimpedance index (height^2^/impedance) were the independent variables. The condition for probability F-to-enter was ≤0.05, with probability F-to-remove ≥0.10. The Pearson product-moment correlation coefficient was used to measure the strength of the linear relationship between BIA- and DXA-estimated FFM. A Bland–Altman plot was further used to measure the agreement between BIA and DXA. The limits of agreement (LOA) were set to mean ± 2 SD [13].

Male (*n* = 403) and female (*n* = 301) participants were divided into three separate groups of *n* = 134, 134, and 135 and *n* = 100, 100, and 101, respectively. Two-thirds of the participants were used as the modeling group to construct the FFM^m^_HH_, FFM^m^_HF_ and FFM^f^_HH_, FFM^f^_HF_ estimate models. Split-sample analysis was used for cross-validation by randomly assigning participants into the modeling group using regression analysis to construct the prediction model. The other 1/3 of the participants were used as the validation group to calculate the correlation, LOA, and pure error (PE) between FFM_HH_, FFM_HF_, and FFM_DXA_, using correlation and Bland–Altman analyses. The research method for the validation group was based on that by Macis et al. [14]. Different groupings were used separately to construct and validate the FFM_HH_ and FFM_HF_ regression models.

One-factor ANOVA was used to compare the difference between two mean values. A confidence interval level of 5% (*p* < 0.05) was considered significant. All data were analyzed using SPSS. 17.0 software (SPSS Inc., Chicago, IL, USA, 2008).

## 3. Results

A total of 704 Asian subjects (403 men and 301 women) participated (Table 1). Men and women were aged 33.1 ± 17.0 years and 37.5 ± 16.1 years, respectively, with body fat percentages of 20.9% ± 8.9% and 33.7% ± 9.5%, respectively.

**Figure 3:**
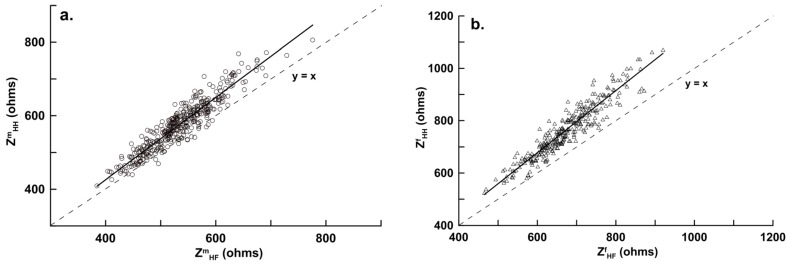
Correlations between Z_HF_ (hand-to-foot impedance) with Z_HH_ (hand-to-hand impedance) for (**a**) male subjects (*n* = 403; Z^m^_HH_ = 1.119, Z^m^_HF_ − 22.065, *r* = 0.938, SEE = 24.229 ohms, *p* < 0.001); and (**b**) female subjects (*n* = 301; Z^f^_HH_ = 1.186, Z^f^_HF_ − 33.970, *r* = 0.943, SEE = 34.021 ohms, *p* < 0.001). SEE, standard error of the estimate; *r*, coefficient of correlation.

The within-day CV% for hand-to-foot (whole body) and hand-to-hand model impedance for the six subjects were 0.2%–0.7% and 0.3%–0.9%, respectively. The between-day CV% for the same subjects and the same measurement was 0.9%–1.8% and 1.0%–2.2%, respectively. The scatter plot and linear regression impedance values between hand-to-hand (Z^m^_HH_) and hand-to-foot (Z^m^_HF_) for male participants are shown in Figure 3a, and are shown in Figure 3b for female participants. The slopes and intercepts were different (*p* < 0.05) from one and zero, respectively, for both sexes.

During the development of the predictive equations, the variance inflation factors (VIFs) in the FFM estimate equations for both the hand-to-foot (FFM^m^_HF_, FFM^f^_HF_) and hand-to-hand (FFM^m^_HH_, FFM^m^_HH_) models for both male and female participants were <3, indicating no collinearity between the equations; however, when sex was considered, the VIF for the h^2^/Z estimate variable was >5.0 (Table 2). In the stepwise multiple regression analysis, the important estimating variables were automatically and sequentially included based on importance (Table 2), resulting in h^2^/Z, age, and body weight as the important of a significant (*) variables regardless of hand-to-foot or hand-to-hand model.

**Figure 4:**
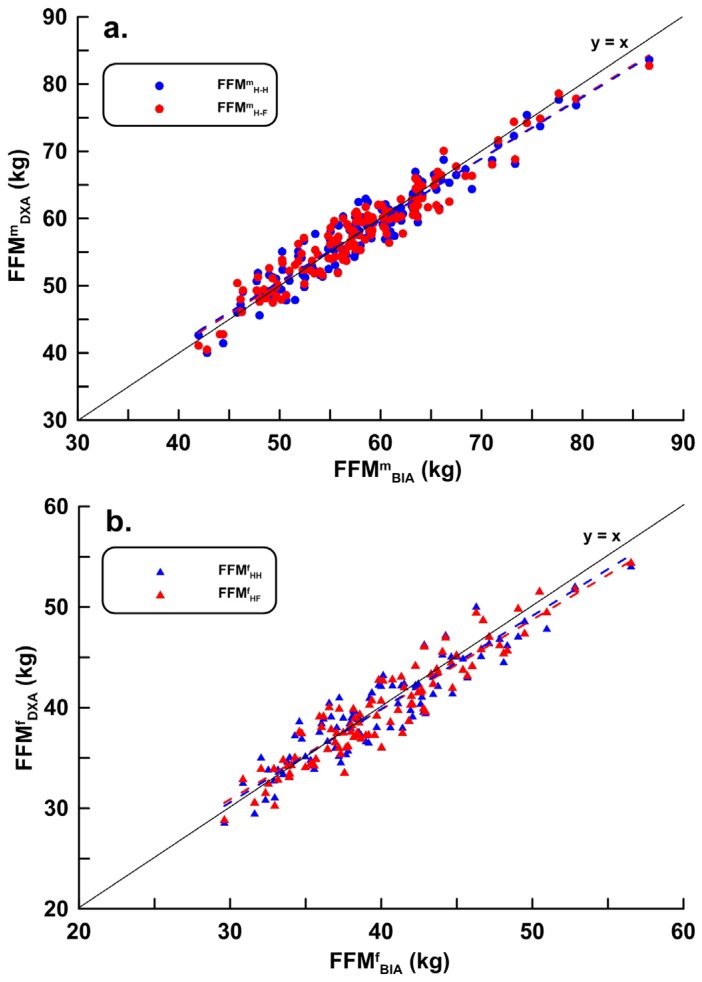
Scatter plots and regression lines for the validation of FFM_HH_ and FFM_HF_ for (**a**) male subjects (*n* = 134; FFM^m^_HH_: *y* = 0.982 *x* + 0.996, *r*^2^ = 0.926, SEE = 2.233 kg , *p* < 0.001; FFM^m^_HF_: *y* = 1.011*x* − 0.577, *r*^2^ = 0.925, SEE = 2.181 kg, *p* < 0.001); and (**b**) female subjects (*n* = 100; FFM^f^_HH_: *y* = 1.054*x* − 2.204, *r*^2^ = 0.870, SEE = 1.937 kg, *p* < 0.001; FFM^f^_HF_: *y* = 1.034*x* − 1.267, *r*^2^ = 0.891, SEE = 1.776 kg, *p* < 0.001).

**Figure 5:**
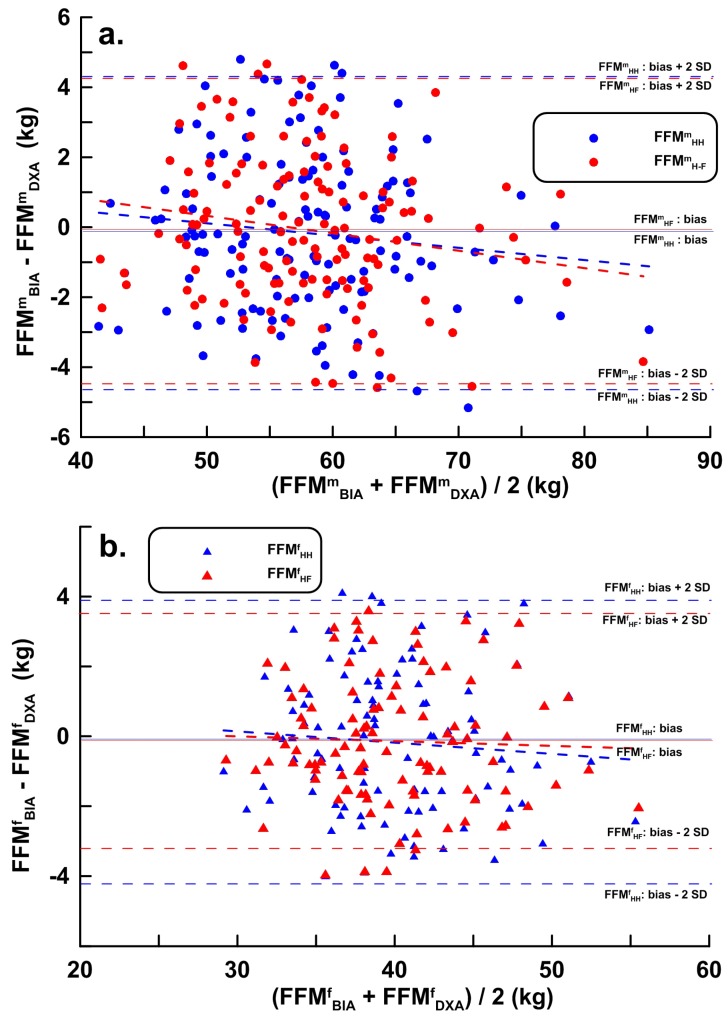
Scatter plots and regression lines for the validation of fat free mass (FFM_HH_ and FFM_HF_) estimated using dual-energy X-ray absorptiometry or bioelectrical impedance analysis (BIA) for (**a**) male subjects (*n* = 134; FFM^m^_HH_: bias = −0.164 kg, LOA: −4.616–4.289 kg, *y* = 1.865 − 0.031*x*, *r* = 0.118, *p* = 0.173; FFM^m^_HF_: bias = −0.070 kg, LOA: −4.395–4.255 kg, *y* = 2.613 − 0.043*x* (*r* = 0.127, *p* = 0.062); and (**b**) female subjects (*n* = 100; FFM^f^_HH_: bias = −0.178 kg, LOA: −4.271–3.916 kg, *y* = 1.103 − 0.032*x*, *r* = 0.079, *p* = 0.434; FFM^f^_HF_: bias = −0.137 kg, LOA: −3.750–3.476 kg, *y* = 0.409 − 0.014*x*, *r* = 0.038, *p* = 0.703).

Estimated FFM Equations (1a), (1b), (2a) and (2b) were obtained using the modeling group data (men, *n* = 269; women, *n* = 201). Validation data (men, *n* = 134; women, *n* = 100) were substituted into the estimated equations, and the correlation coefficients, LOA, PE, and bias between FFM_HH_, FFM_HF_, and FFM_DXA_ are shown in Table 3. The distribution and regression lines for male FFM^m^_HH_ and FFM^m^_HF_ and female FFM^f^_HH_, FFM^f^_HF_, and FFM_DXA_ are shown in Figure 4a,b, with corresponding Bland–Altman analyses in Figure 5a,b.

In Figure 3a,b, the intercepts of the regression lines were −22.065 (95% confidence interval (CI), −43.085, −1.045) and −33.970 (95% CI, −64.201, −3.739), the slopes were 1.119 (95% CI, 1.014, 1.224) and 1.186 (95% CI, 1.013, 1.459); the 95% CIs for both the intercept and slope did not cross 0 or 1 when referring to the ordinary least products regression analysis determination of the correlation value for the proportional bias and fixed bias [15], although the male and female hand-to-foot and hand-to-hand impedance values were positively correlated but were not interchangeable. In Figure 4, the intercepts of the regression lines for the hand-to-hand and hand-to-foot BIA models were 0.996 (95% CI, −0.526, 2.518) and −0.577 (95% CI, −2.100, 0.946), respectively, and the slopes were 0.982 (95% CI, 0.670, 1.294) and 1.011 (95% CI, 0.955, 1.067), respectively. In Figure 4b, the intercepts of the regression lines for women were −2.204 (95% CI, −4.636, 0.228) and −1.267 (95% CI, −2.612, 0.078), respectively, and the slopes were 1.054 (95% CI, 0.986, 1.121) and 1.034 (95% CI, 0.967, 1.101), respectively. The 95% CIs of the intercepts and slopes of the four regression equations crossed 0 and 1, indicating no proportional bias and fixed bias (unbiased and precise) from the two hand-to-hand and hand-to-foot BIA models.

The correlation coefficients between FFM^m^_HH_ and FFM_DXA_ for men for the three validation analyses were 0.963, 0.960, and 0.952 with LOAs of −4.249–4.353 kg, −4.596–3.812 kg, and −4.261–4.873 kg, respectively. The correlation coefficients for FFM^m^_HF_ were 0.962, 0.961, and 0.952, with LOAs of −4.402–4.296 kg, −4.538–3.773 kg, and −4.127–5.056 kg, respectively. The correlation coefficients for FFM^f^_HH_ for women were 0.933, 0.933, and 0.921, with LOA −3.843–3.936 kg, −3.562–3.769 kg, and −4.236–3.953 kg. The correlation coefficient for female participants’ FFM^f^_HF_ were 0.944, 0.944, and 0.939, with LOAs of −3.646–3.457 kg, −3.157–3.545 kg, and −3.730–3.476 kg, respectively.

## 4. Discussion

FFM using the newly developed hand-to-hand BIA model conducted in the standing position was highly correlated with the FFM measured using DXA. We used changeable electrodes connected to the same instrument measuring the hand-to-foot and hand-to-hand impedances in a standing position. The hand-to-hand and hand-to-foot BIA model results were not only highly correlated for both male and female participants, but also reliable for measuring FFM, based on the cross-validation and LOA range of the two models.

Similar BIA results from both supine and standing positions were reported by Demura et al. [16]; the author used four different models to estimate the results from hand-to-hand and hand-to-foot BIA, and all four measurements were highly correlated. However, the study was conducted with a much smaller sample of men, with a narrower age range (20 years). In addition, the data obtained using segmental BIA method in the supine position, as reported by Organ et al., were highly correlated with the body fat data measured using the underwater weighing method [17]. According to our results, the estimation of BF% using the hand-to-hand BIA model in the standing position was strongly correlated with DXA-estimated body fat. Furthermore, the accuracy of the hand-to-hand BIA model results were relatively greater than those obtained using hand-to-foot BIA model, suggesting that hand-to-hand BIA model in the standing position is feasible for the evaluation of body composition.

Additionally, the h^2^/Z^m^_HF_ and h^2^/Z^m^_HH_ estimate variance represented 90.1% and 90.2% of the variability, respectively, in the entire FFM estimation equation for male participants, compared with 80.0% and 85.4% of the variability, respectively, for female participants. Regardless of sex, the current hand-to-foot BIA model provided a better estimate than the hand-to-hand BIA model. Because a high correlation between the results of the two methods cannot completely ensure agreement between the two methods [13], we used Bland–Altman analysis; the LOA indicated that the hand-to-foot BIA model is more accurate than the hand-to-hand BIA model, regardless of sex. In a previous study of a BIA FFM estimation model for Chinese participants, the LOAs were −7.2–6.0 kg in men and −4.0–6.4 kg in women, based on a comparison with FFM obtained using an underwater weighing method [18,19]. The LOAs of the hand-to-hand BIA model in the present study were −4.616–4.289 kg and −4.271–3.916 kg for men and women, respectively. Upon direct observation of the LOA region, the region was wider and could be applied in epidemiological or clinical Geneva BIA equations [20]. Although the results of the Geneva BIA equation published by Kyle et al. were satisfactory, with a high *r*^2^ (0.96) and low SEE (LOA = −3.4–3.5 kg and SEE = 1.72 kg), the BMI range was narrower (17.0–33.8 kg/m^2^) than that in the present study (15.9–43.1 kg/m^2^). Hence, the hand-to-hand BIA model constructed in the present study has a wider and more reasonable range that can be applied to a larger population.

Houtkooper et al. [21] reported a subjective rating system for the evaluation of SEE equations used to predict FFM in adults, which was adapted and summarized in Table 1. In the present study, the predictive ability of the hand-to-hand BIA model was rated as ideal for men (SEE = 2.0–2.5 kg) and excellent to very good for women (SEE = 1.8–2.3 kg). Our results were consistent with the findings of Houtkooper et al., and are of value for predicting FFM. h^2^/Z, age, sex, and weight were important variables for the hand-to-foot BIA model, with correlation coefficients with FFM_DXA_ of 0.957, 0.370, 0.807, and 0.776, respectively. When sex was excluded from the hand-to-hand and hand-to-foot models, the order of importance was h^2^/Z, age, and weight. In male participants, the hand-to-foot BIA model correlation coefficients were 0.901, 0.471, and 0.797. In addition, age had the lowest correlation with FFM_DXA_, but it was the second selected variable in the stepwise method. In the present study, the utilization of three separate groupings (2/3 of the participants as the modeling group and 1/3 of the participants as the validation group) were used to construct and validate the FFM_HH_ and FFM_HF_ regression models confirmed the reliability and accuracy of both the hand-to-hand and hand-to-foot BIA models for assessing FFM.

Because sex could play an important role in predicting FFM using BIA measurements, separate predictive equations were developed for men and women. Due to the high correlation and lower SEE values, the equations for Z_HF_ performed better than Z_HH_ for predicting FFM in men. This was not true for female participants. These results are similar to those reported by Sun et al. [22]. The differences between the sexes may be attributable to body fluids, visceral fat, and menstrual status in women [23,24].

A study conducted by Organ et al. [17] indicated that the proportion of the resistance value among the arm, trunk, and leg were 13.8:1.0:11.8. The arm impedance value only accounts for half of the impedance from the hand-to-foot BIA model. Furthermore, the results from the current study showed that the hand-to-hand and hand-to-foot model impedance values are highly correlated; after cross-validation of the FFM results, both the hand-to-hand and hand-to-foot BIA models also demonstrated high reliability.

Although the impedance composition is low in the trunk area, a high CV% value in the hand-to-foot mode would affect the impedance results. Further, the passage of current through the trunk area was different with the hand-to-hand model than with the hand-to-foot model. Further research is needed to explain this phenomenon.

For BIA FFM estimation equations, some researchers use the impedance as the estimating variable; for example, the Z(impedance) has been used as the estimating variable for standing foot-to-foot BIA FFM estimating equations [25] and in a similar standing position BIA body composition estimation equation model [13]. Furthermore, resistance or impedance has been used as different estimating variables to estimate FFM, total body water, and total body potassium, with no significant difference between the two methods [26,27]. In the study by Hannan et al. [28], the validity and reliability of multiple frequency impedance analysis to estimate FFM were similar to that of the single 50 kHz frequency used in the present study. Further, consensus regarding the superiority of single or multiple impedance frequency is lacking [29,30].

Although the present sample included only healthy adults, the distribution of body fluid can change with gravity and different postures [31]. Changes in impedance between supine and standing positions for hand-to-foot BIA measurements indicate that gravity has a minor effect on body fluid [32]. Regardless of the standing posture BIA model (hand-to-foot or hand-to-hand), the impedance values would change based on the time spent performing the measurements; the impedance using the hand-to-hand model would increase to approximately 30 ohms throughout the day [33]. When using a hand-to-hand BIA model to measure body composition, a well-controlled environment and individual factors have to be considered [34].

The results of the current study are not likely generalizable to Caucasians, African Americans, extremely obese individuals, or extremely underweight individuals. Furthermore, DXA is not reliable for over- or under-hydrated subjects. In the present study, DXA was used as a criterion measurement to derive the prediction model. Both accurate and inaccurate body composition results have been reported for larger body sizes with DXA [35,36]. Further research is needed to identify the cause of measurement errors with DXA, such as thickness, hydration status, soft tissue content attached to the bones alone, and fat distribution. Finally, subject size, calibration procedures, software versions, and machine model and manufacturer would all affect the accuracy of DXA measurements, and should be considered when conducting experiments [37].

Hand-to-hand BIA model measuring methods have been widely used in epidemiological studies and individual body composition measurements. The accuracy and reliability reported from the present study indicate that the hand-to-hand BIA model method is a convenient method for group measures. Further research is recommended to investigate the application of BIA estimation methods for body composition in individuals.

## 5. Conclusions

In summary, the hand-to-hand BIA model for the estimation of body composition produces similar results to the existing DXA and hand-to-foot BIA model measurements. The hand-to-hand BIA model is a convenient and practical application for FFM assessment that may be applied in epidemiological studies for the measurement of body composition in a healthy Chinese population.

## Figures and Tables

**Table 1:** General physical characteristics and body composition data for male and female subjects.

Total	Mean ± SD	Range	Mean ± SD	Range	*p*
	Male (*n* = 403)		Female (*n* = 301)		
Age (years)	33.1 ± 17.0	17.0–81.5	37.5 ± 16.1	17.1–77.6	<0.01
Height (cm)	172.5 ± 7.5	151.5–200.0	159.6 ± 6.8	142.5–178.0	<0.001
Weight (kg)	74.0 ± 13.1	42.0–133.8	60.0 ± 12.2	38.0–108.3	<0.001
BMI (kg/m^2^)	24.8 ± 3.7	16.8–41.8	23.6 ± 4.5	15.8–42.7	<0.05
**DXA**					
FFM_DXA_ (kg)	58.5 ± 8.1	35.6–92.9	39.9 ± 5.5	28.4–58.7	<0.001
BF%_DXA_ (%)	20.9 ± 8.9	5.1–41.5	33.7 ± 9.5	10.4–56.6	<0.001
**Bioimpedance**					
Z_5,HF_ (Ω)	594.1 ± 60.3	423.3–834.3	763.4 ± 107.7	512.6–982.2	<0.001
Z_5,HH_ (Ω)	654.4 ± 76.4	450.3–852.0	860.9 ± 83.2	355.5–1103.4	<0.001
Z_50,__HF_ (Ω)	533.4 ± 58.6	384.3–776.0	673.3 ± 80.8	465.0–920.0	<0.001
Z_50,H__H_ (Ω)	576.3 ± 69.9	409.7–806.0	764.8 ± 101.7	525.0–1071.0	<0.001
Z_100HF_ (Ω)	482.7 ± 55.3	261.2–743.0	632.4 ± 95.1	434.1–888.3	<0.001
Z_100,HH_ (Ω)	532.9 ± 79.2	366.5–703.0	715.8 ± 95.2	394.3–943.4	<0.001
R_50,HF_ (Ω)	529.3 ± 58.2	380.2–774.0	669.2 ± 98.9	461.7–916.3	<0.001
R_50,HH_ (Ω)	562.1 ± 60.3	386.7–740.3	754.3 ± 99.5	452.3–1005.6	<0.001
X_50,HF_ (Ω)	64.3 ± 7.5	33.2–110.1	73.1 ± 8.9	42.5–114.5	<0.001
X_50,HH_ (Ω)	86.5 ± 8.9	49.4–131.8	103.2 ± 12.3	47.5–176.4	<0.001
PhA_50,HF_ (deg)	6.9 ± 0.8	4.1–9.2	6.2 ± 0.7	4.2–8.8	<0.001
PhA_50,HH_ (deg)	8.7 ± 1.1	6.0–10.8	7.8 ± 1.2	4.5–9.7	<0.001
**BIA**					
FFM_HF_ (kg)	58.5 ± 7.1 ^&^	39.0–86.6	39.9 ± 4.9 ^&^	29.1–56.2	<0.001
FFM_HH_ (kg)	58.5 ± 7.5 ^#^^,&^	37.9–89.3	39.9 ± 4.8 ^#^^,&^	28.9–54.6	<0.001
**Modeling group**	**Male (*n* = 269)**		**Female (*n* = 201)**		
Age (years)	32.6 ± 16.5	17.2–78.8	37.3 ± 16.2	17.1–74.8	<0.01
Height (cm)	172.6 ± 7.4	153.0–200.0	159.7 ± 6.8	142.5–176.0	<0.001
Weight (kg)	74.1 ± 13.9	42.0–133.8	59.9 ± 11.9	38.0–107.4	<0.001
BMI (kg/m^2^)	24.8 ± 3.6	16.8–39.9	23.5 ± 4.5	15.8–37.8	<0.05
**DXA**					
FFM_DXA_ (kg)	58.7 ± 7.6	35.6–87.4	39.9 ± 5.2	28.4–58.7	<0.001
BF%_DXA_ (%)	20.9 ± 8.7	5.1–41.0	33.5 ± 9.4	13.5–56.6	<0.001
**Bioimpedance**					
Z_50,__HF_ (Ω)	530.4 ± 59.0	384.3–776.0	674.7 ± 84.6	465.0–920.0	<0.001
Z_50,__HH_ (Ω)	572.2 ± 69.7	409.7–806.0	766.1 ± 106.1	525.0–1071.0	<0.001
**BIA**					
FFM_HF_ (kg)	58.7 ± 7.0 ^&^	39.0–86.6	39.9 ± 4.8 ^&^	29.1–56.2	<0.001
FFM_HH_ (kg)	58.8 ± 7.4 ^#,&^	37.9–89.3	40.0 ± 4.8 ^#,&^	29.5–54.6	<0.001
**Validation Group**	**Male (*n* = 134)**		**Female (*n* = 100)**		
Age (years)	34.0 ± 18.0	15.3–81.5	38.0 ± 16.1	17.2–77.6	<0.01
Height (cm)	172.5 ± 7.6	151.5–193.0	159.4 ± 6.8	143.0–178.0	<0.001
Weight (kg)	73.9 ± 13.5	52.0–131.0	60.3 ± 12.8	42.0–108.3	<0.001
BMI (kg/m^2^)	24.8 ± 3.8	18.7–41.8	23.7 ± 4.4	17.4–42.7	<0.05
**DXA**					
FFM_DXA_ (kg)	58.0 ± 7.7	42.0–86.6	40.0 ± 5.2	28.9–56.5	<0.001
BF%_DXA_ (%)	21.1 ± 9.4	5.1–41.0	34.2 ± 9.5	9.4–54.3	<0.001
**Bioimpedance**					
Z_50,__HF_ (Ω)	539.2 ± 57.4	407.7–729.0	670.7 ± 73.1	517.3–856.5	<0.001
Z_50,__HH_ (Ω)	584.4 ± 69.7	439.3–769.0	762.0 ± 92.8	583.0–1035.5	<0.001
**BIA**					
FFM_HF_ (kg)	57.9 ± 7.4 ^&^	40.5–82.8	39.8 ± 5.1 ^&^	29.0–54.5	<0.001
FFM_HH_ (kg)	57.8 ± 7.5 ^#^^,&^	40.0–83.7	39.8 ± 5.1 ^#,&^	28.6–54.1	<0.001

Values are reported as range (minimum–maximum). Significantly different from male subjects (one-factor ANOVA), *p* < 0.05, 0.01, 0.001. Mean values were not significantly different from those for FFM_DXA_: ^&^ *p* > 0.05. Mean values were not significantly different from those for FFM_HF_: ^#^ *p* > 0.05. DXA, dual energy X-ray absorptiometry; FFM, fat free mass; BF%, body fat percentage; BMI, body mass index; FFM_DXA_, DXA-measured fat free mass; BF%_DXA_, DXA-measured percentage body fat; FFM_HF_, hand-to-foot model-estimated fat free mass; FFM_HH_, hand-to-hand-model estimated fat free mass; BF%_HF_, hand-to-foot model-estimated percentage body fat; BF%_HH_, hand-to-hand model-estimated percentage body fat; Z_5_, impedance at 1 kHz; Z_50_, impedance at 50 kHz, Z_100_, impedance at 100 kHz; R_50_, Resistance at 50 kHz; Xc_50_, Reactance at 50 kHz; PhA_50_, R_50_, resistance at 50 kHz, X_50_, reactance at 50 kHz, Phase Angle at 50 kHz; subscripts HH and HF represent hand-to-hand and hand-to-foot models, respectively.

**Table 2:** Multiple regression analysis results for FFM_DXA_, based on BIA-measured h^2^/Z_HF_ and h^2^/Z_HH_ Total subjects, *n* = 704; Male subjects (Modeling group, *n* = 269); Female subjects (Modeling group, *n* = 201).

h^2^/Z	Age	Weight	Gender	Intercept	SEE (kg)	*r*^2^	VIF	β
FFM_HH_, Total subjects (*n* = 704)								
0.948 ± 0.011 *	-	-	-	8.273 ± 0.500 *	3.304	0.915	6.18	0.56
0.912 ± 0.009 *	−0.107 ± 0.006 *	-	-	13.629 ± 0.524 *	2.805	0.939	1.10	−0.19
0.784 ± 0.013 *	−0.118 ± 0.006 *	0.131 ± 0.101 *	-	10.813 ± 0.527 *	2.520	0.951	2.68	0.23
0.556 ± 0.017 *	−0.131 ± 0.005 *	0.184 ± 0.009 *	4.967 ± 0.293 *	14.955 ± 0.507 *	2.124	0.965	3.29	0.22
FFM_HF_, Total subjects (*n* = 704)								
0.962 ± 0.010 *	-	-	-	3.562 ± 0.515 *	3.092	0.926	6.46	0.58
0.927 ± 0.009 *	−0.096 ± 0.006 *	-	-	8.626 ± 0.552 *	2.670	0.945	1.36	−0.18
0.813 ± 0.014 *	−0.122 ± 0.005 *		3.233 ± 0.315 *	12.585 ± 0.643 *	2.491	0.952	3.18	0.22
0.583 ± 0.017 *	−0.122 ± 0.005 *	0.163 ± 0.009 *	5.058 ± 0.281 *	16.917 ± 0.800 *	2.072	0.967	2.92	0.21
FFM^m^_HH_, Male subjects (*n* = 269)								
0.881 ± 0.026 *	-	-	-	12.062 ± 1.389 *	3.304	0.812	2.41	0.55
0.816 ± 0.024 *	−0.103 ± 0.011 *	-	-	18.864 ± 1.413 *	2.873	0.858	1.36	−0.27
0.537 ± 0.026 *	−0.126 ± 0.009 *	0.217 ± 0.015 *	-	18.235 ± 1.066 *	2.164	0.919	2.22	0.37
FFM^m^_HF_, Male subjects (*n* = 269)								
0.870 ± 0.026 *	-	-	-	9.168 ± 1.465 *	3.285	0.813	2.49	0.55
0.806 ± 0.023 *	−0.103 ± 0.011 *	-	-	16.151 ± 1.482 *	2.862	0.859	1.14	−0.27
0.532 ± 0.027 *	−0.126 ± 0.009 *	0.213 ± 0.016 *	-	16.663 ± 1.144 *	2.208	0.916	2.29	0.36
FFM^f^_HH_, Female subjects (*n* = 201)								
0.821 ± 0.044 *	-	-	-	12.089 ± 1.495 *	3.138	0.640	2.08	0.60
0.836 ± 0.030 *	−0.140 ± 0.019 *	-	-	16.778 ± 1.082 *	2.172	0.828	1.05	−0.45
0.615 ± 0.038 *	−0.144 ± 0.008 *	0.132 ± 0.016 *	-	16.507 ± 0.939 *	1.884	0.870	2.08	0.30
FFM^f^_HF_, Female subjects (*n* = 201)								
0.831 ± 0.036 *	-	-	-	8.020 ± 1.389 *	2.720	0.730	2.31	0.67
0.818 ± 0.025 *	−0.121 ± 0.008 *	-	-	13.036 ± 1.024 *	1.890	0.869	1.02	−0.40
0.651 ± 0.035 *	−0.128 ± 0.008 *	0.100 ± 0.016 *	-	13.675 ± 0.940 *	1.724	0.891	2.32	0.23

FFM_DXA_, DXA-measured fat free mass; Regression coefficient estimate ± SEE; *r*^2^, coefficient of determination; * *p* < 0.001; β, standardized coefficient; VIF, variance inflation factor; SEE, standard error of the estimate.

**Table 3:** Prediction equation for the validation group.

Modeling Group	Male (*n* = 269)	
Measured FFM_DXA_	58.7 ± 7.6 kg	
Validation Group	Male (*n* = 134)	
Measured FFM_DXA_	58.0 ± 7.7 kg	
Prediction FFM^m^_HH_	0.537 h^2^/Z^m^_HH_ − 0.126 yr + 0.217 w + 18.235, (*r*^2^ = 0.919, SEE = 2.164 kg, *n* = 269)	(1a)
Using Validation group FFM^m^_HH_	57.8 ± 7.5 kg, *r* = 0.957, LOA = −4.616–4.289 kg, PE = 2.224 kg, bias = −0.164 kg	
Prediction FFM^m^_HF_	0.532 h^2^/Z^m^_HF_ − 0.126 yr + 0.213 w + 16.663, (*r*^2^ = 0.916, SEE = 2.208 kg, *n* = 269)	(1b)
Using Validation group FFM^m^_HF_	57.9 ± 7.4 kg, *r* = 0.960, LOA = −4.395–4.255 kg, PE = 2.156 kg, bias = −0.070 kg	
**Modeling Group**	**Female (*n* = 201)**	
Measured FFM_DXA_	39.9 ± 5.2 kg	
Validation Group	Female (*n* = 100)	
Measured FFM_DXA_	40.0 ± 5.2 kg	
Prediction FFM^f^_HH_	0.615 h^2^/Z^m^_HH_ − 0.144 yr + 0.132 w + 16.507, (*r*^2^ = 0.870, SEE = 1.884 kg, *n* = 201)	(2a)
Using Validation group FFM^f^_HH_	39.8 ± 5.1 kg, *r* = 0.921, LOA = −4.271–3.916 kg, PE = 2.044 kg, bias = −0.178 kg	
Prediction FFM^f^_HF_	0.651 h^2^/Z^m^_HH_ − 0.128 yr + 0.100 w + 13.675, (*r*^2^ = 0.891, SEE = 1.724 kg, *n* = 201)	(2b)
Using Validation group FFM^f^_HF_	39.8 ± 5.1 kg, *r* = 0.939, LOA = −3.750–3.476 kg, PE = 1.803kg, bias = −0.137 kg	

FFM, fat free mass; DXA, dual energy X-ray absorptiometry; FFM_DXA_, DXA-measured FFM; FFM_BIA_, BIA-predicted FFM; h^2^/Z, height^2^/impedance; subscripts HH and HF represent hand-to-hand and hand-to-foot models, respectively; superscripts f and m represent female and male participants; SEE, standard error of the estimate; LOA, limits of agreement, bias: average of difference; yr, age; w, weight; *r*, correlation coefficient; *r*^2^, determinate coefficient; PE, pure error = $\sqrt{\sum^{\;}\frac{{\left(y^{\prime }-y_{i}\right)}^{2}}{n}}$, where y’ the prediction FFM, *y* is the observed, and *n* is the number of subjects.
